# Hospital Meetings, &c.

**Published:** 1899-02-18

**Authors:** 


### HOSPITAL MEETINGS, &C.

### CITY OF LONDON TRUSS SOCIETY.

Mr. John Norbury, the treasurer, presided at the annual general meeting of the City of London Truss Society, at the offices, 35, Finsbury Square, on Wednesday (1st inst.) From the accounts presented it appeared that the ordinary receipts, with the bank balance brought forward of £885, and including £749 from the festival, amounted to £4,804; on the same side of the balance-sheet appearing £790 in legacies and £1,600 in loans. The ordinary expenditure, including bank balance £190, amounted to £6,245, with £950 investments added. The report read by the secretary, Mr. John Whittington, pointed out that while the annual subscriptions showed an increase of £60 10s. over those of 1897, it was a matter of regret that the life subscriptions and donations showed a falling off to the extent of £147. The sum received on account of legacies was £722 less than in the previous year. The total income from all sources was thus £4,709 18s. 9d., as against £5,551 9s. 3d. in 1897. The patients relieved in 1898 numbered 9,476, making a total since the foundation of the society in 1807 of 539,358, the ages of the patients ranging from three weeks to 95 years. The number of instruments supplied was 9,551. The report was agreed to without discussion, and after the usual votes of thanks to the auditors, stockbrokers, secretary, and chairman, the meeting was made special, and the sanction of the governors given to sell out £3,000 of the funded property to pay off the various loans, and so save the interest being paid. Much regret was expressed at the necessity for adopting this course, which it was hoped would not have to be repeated, and special stress was laid on the fact that nothing was received by the society from either the Prince of Wales's nor the Hospital Saturday or Sunday Funds, it not coming within their schedules as a hospital.

### THE AFTER-CARE ASSOCIATION.

The annual meeting of the "After-Care Association for Poor Persons Discharged Recovered from Asylums for the Insane" was held, by permission of Sir Samuel Wilks, Bart., at 72, Grosvenor Street, W., on the 6th inst. Sir Samuel Wilks took the chair at three o'clock, and the Secretary read the report for 1898. It stated that 186 cases, of which 131 were women and 55 men, had passed before the council, as compared with 147 during the previous twelve months; whilst some few cases had been refused as they were not fitted for the struggle of life again. Considerable initial expense is incurred by the thorough investigation that is made by the society's permanent official, not only of each case, but of the situations and employers to whom they are sent. This has been fully justified by the satisfactory way in which the majority have retained their situations, and by the comparatively few failures amongst them. Some cases, not coming under the usual headings, have been assisted in other ways. Convalescents are boarded out in cottage homes, which are under the personal inspection of the secretary. They are mostly in the South of England, and additional homes are much needed. The council have inquired of the various Boards of Guardians as to the cases of workhouse inmates discharged from asylums in order that the society might undertake the charge of suitable cases. Several Boards have consented to furnish information on this point, but the inquiry is too recent to afford any data by which to estimate its success. Some of the London Boards of Guardians have become subscribers to the funds of the association, and a large number of district secretaries have been appointed. The past year has been the most successful in the annals of the association, and thanks are due to several of the City companies for contributions, to Mrs. Kendzior, who gave an entertainment; and to Dr. and

Mrs. Bower, who held a meeting on its behalf. The income amounted to £561, and there is a slight balance in hand. Sir Samuel Wilks then expressed his pleasure in assisting this important association. Philanthropists, he said, provided convalescent homes, societies to help those injured by accident, fire, or shipwreck. But there were other troubles much more intimate and private that needed help in a more delicate and tactful way, and a poor person discharged from an asylum was much more desolate than a shipwrecked sailor. He could imagine how a servant entering a new place, a business woman harrowed by money troubles, or the grief of a misplaced affection, might be thrown off their mental balance. With a few months' care they were cured, but they could not return to their friends or to their work. They must not mention their "last place," for that would cause everyone to shun them. It was for these poor people that this association cared, and it needed money to find them new homes and employment. The Bishop of Islington moved the report, and said that the need of the society's work was obvious. It only needed to be stated in order to appeal to the intellect and sympathies of philanthropists, and the particular work of the association needed only to be brought vividly before the minds of the public to receive the support it merited. Dr. Blandford, the Archdeacon of Essex, and Dr. Savage supported the resolution. The first testified to the value of intermediate treatment between the asylum and the return home in cases amongst the well to do classes; the second to the help this association had been amongst the poor in his own district in East London; and the last described the methods in which aid was given. He dwelt also upon the dangers of convalescence. "Then the mind," he said, "was unstable, deep gloom often succeeded the fit of mental aberration, whilst a return to the circumstances which caused the downbreak was likely to cause a relapse. Each relapse predisposed to another, and tended to establish a habit of recurrence and so militated against the chances of permanent cure." All speakers urged the importance of disabusing the public mind of the fallacy that insanity was incurable. It was perfectly curable in many instances, and even in cases of recurrent periods of insanity the patient during the intervals enjoyed absolute mental health. The election of the officers of the council (to whose number were added Sir Samuel Wilks and the Bishop of Islington as vice-presidents, and Dr. Savage as a member of the council) was moved by Dr. Nicholson and seconded by Dr. Shaw. The Rev. Henry Hawkins proposed the vote of thanks to the chairman, which was seconded by Dr. Rayner. Tea was served to those present at the end of the proceedings.

### ST. MARK'S HOSPITAL.

At the Mansion House on Thursday, the 9th inst., Mr. Geo. Anson Whealler presided at the annual meeting of St. Mark's Hospital for Fistula and Other Diseases of the Rectum, in the absence of the Lord Mayor—who has consented to be president of the hospital during his Mayoralty—and of Mr. Biddulph Martin, the treasurer, both of whom were prevented by indisposition from being present. The sixty-third annual report, which was adopted without discussion on the motion of the Chairman, pointed out that the usefulness of the institution had increased during the past year, the number of in-patients (349) having increased by 40, and the out-patients (995) by 109, as compared with the figures of the previous year. The ordinary income was £4,986, plus £804 received in legacies, and the ordinary expenditure £3,429. During the year outstanding accounts amounting to £594 were paid, and £1,020 spent in the purchase of Hotel Cecil Debenture Stock on account of the Julia Henty Fund for the endowment of a bed, the bank balance in hand amounting to close upon £2,000, a very satisfactory state of affairs upon which the chairman congratulated the meeting, expressing the hope that they had at length got into smooth water. The festival dinner produced £2,849, and it is intended to repeat this periodically. The committee record with pleasure a special contribution of £1,200 received through the instrumentality of Mr. D. H. Goodsall from the representatives of the late Mr. Thomas Stone, towards the improvements and other matters to which the deceased gentleman had expressed his intention of contributing. Regret and surprise was expressed that no donation had this year been received from the Prince of Wales's Fund. The secretary, Mr. Edgar Penman, stated that he had written inquiring the cause of the omission, and had received from Sir Savile Crossley a reply to the effect that no application had been made. They had, however, received a donation last year from the Fund without having applied for it, and were much disappointed at being left in the cold, especially as they knew of many subscriptions and donations they would have received had the Fund not absorbed them. Mention was made of the fact that Mr. Alfred Cooper was resigning his post as honorary surgeon, having reached the age limit allowed by the rules, and the customary votes of thanks brought the proceedings to a close.

### LONDON FEVER HOSPITAL.

Lord Balfour of Burleigh presided at the annual general meeting of the London Fever Hospital on Friday last (10th inst), at the Victoria Hotel, Northumberland Avenue. Mr. Hamer, J.P., chairman of the Finance Committee, presented the statement of accounts for the year 1898, which gave the ordinary income as £10,901, and the ordinary expenditure as £10,702. Legacies amounting to £3,174 were received, while extraordinary expenditure on repairs, building improvements, and purchase of stock (net £2,011) totalled £4,021. The annual subscriptions were £4,481, an increase of £80 over those in 1897, and the donations were £2,304, an increase of £676. This statement, he thought, might be considered a very satisfactory one. Their rebuilding operations had been completed so far as the committee had sanctioned them, and they now proposed to consider what recommendations should be made as to completion. All the work done so far had been paid for, though to do so they had been obliged to sell out £1,100 of stock.

Dr. Sidney Phillips, the senior physician, read the report of the committee for the year ended December 31st. This showed that 676 patients came under treatment, 599 being admitted in 1898, 77 having come in previously. Of these 322 were scarlet fever cases, 1 per centage of mortality (1·5 per cent.), being one of the lowest recorded; 59 were cases of measles. In these the mortality was 1·7 per cent. Of rubeola 148 cases were treated; all but three who were in hospital on December 31st went out cured, the mortality being, as usual, nil. Forty-nine cases of diphtheria were treated, and in this disease the percentage of mortality was the same as in the previous year, viz., 12 per cent. Twenty-eight patients were sent in under certificates of infectious fevers who were not so affected, mostly cases of sore throat suspected of being diphtheria. Among the officials of the institution two nurses contracted scarlet fever and two diphtheria; one servant contracted measles; all recovered. No cases of post-scarlatinal diphtheria arose in the wards, the arrangements for immediately separating all patients who, on admission, have diphtheria germs from those having scarlet fever only having worked very satisfactorily. The total number of patients was 31 less than in 1897, and 200 less than in 1896, a decrease which corresponds with a gratifying decrease of scarlet fever throughout the metropolitan area; the notifications under the Public Health Act of 1889 sinking from 25,647 cases in 1896 to 22,848 in 1897, and to 16,894 in 1898. Emphasis is laid by the committee on the fact that the London Fever Hospital stands in a different position from those under the authority of the Metropolitan Asylums Board, which are supported by the rates, it being the only fever hospital in London supported by voluntary contributions and by the payments of the patients treated. Cases of fever, other than small-pox, are admitted at any time provided beds are vacant, as well as cases of measles. All patients admitted contribute towards their maintenance, special payments being charged for private wards. Schools, houses of business, and public institutions are thus relieved of centres of infection; and in this connection it is pointed out that each year many nurses and officials from children's and other general hospitals contract fevers, and 54 such cases were received last year by the London Fever Hospital, which thus forms an important part of the general system of voluntary hospitals in London. Accommodation is also frequently found for cases which the Asylums Board has been unable to take.

The report and accounts were adopted without discussion. The CHAIRMAN then moved the approval of the proceedings of the committee and a vote of thanks for their services. The year, he said, had not been one of great pressure, but of quiet and useful work. They must bear in mind, however, that it was just as necessary to keep everything in a state of efficiency, because they never knew when an outbreak might occur, and it would be impossible to get up an organisation when sudden need arose. They must be ready. The 676 patients treated showed an average of beds occupied of about 60 per day, since each stayed about 37 days. Of these 23 were in the private rooms, paying the full charges for treatment. With regard to the others, in 11 cases the fees had been remitted after inquiry; 54 were cases from other hospitals; 71 were domestic servants; 372 were from the families or employés of tradesmen; 18 from schools; and 15 from the police force or their families. The fact that these were all from among those who were necessarily brought in contact with other people showed the importance of the work of the hospital. Beyond these they had had 46 cases put down as belonging to the professional classes, so that there could be no doubt of their usefulness to the general public as preventing the spread of infection. In no case had they refused to admit anyone suffering either from diphtheria, scarlet fever, measles, or German measles. Dr. Phillips, in his statement, had referred to the fact that 75 per cent. of fever cases were now treated in hospitals, and the proportion was yearly increasing. This was most satisfactory, since there could be no doubt that in many private houses there were no proper facilities for securing successful isolation, and there was thus much greater risk of infection spreading than when the patient was moved to a hospital. He could, of course, sympathise with those who were reluctant to send their friends, and especially children, away from home; but their treatment in the hospital was so successful that it should encourage them to do so. They took every precaution to give the fullest information to relatives as to how the case was going on. The patient could be visited twice a week, and if post cards were supplied they were happy to furnish a daily bulletin. He laid special emphasis on these points because there was great want of knowledge on the part of the general public on the subject.

The motion was adopted, as was also a vote of thanks to the honorary staff. The re-election of Lord Burleigh as president for the ensuing year was carried by acclamation, and a vote of thanks for his conduct in the chair concluded the proceedings.

---

### LIVINGSTONE COLLEGE.

THE annual meeting of the Livingstone College was held at the Memorial Hall, Farringdon Street, on Thursday, February 9th, at a quarter past five p.m. Mr. THEODORE HOWARD took the chair, and four missionaries now studying at the college testified to the great value of a little knowledge at the right time in distant places. The proceedings began by a conversazione in the board-room, and after the guests had adjourned to the library the Secretary read the report. The income and expenditure balanced to within £3 (on the right side of the ledger) of £921. There are eleven students attending the course of instruction—seven resident and four non-resident. The Chairman said, in the course of his remarks, that his daughter had only the other day been explaining to him how to lay a bed-room floor in a sanitary manner, and what to do in a case of a street accident; and if a knowledge of the laws and principles of hygiene were necessary in a country where a doctor's brass plate could be seen from every house, how much more necessary was it for those who were going far from all medical assistance. Mr. E. J. Butler, of the Friends' Mission in India, gave detailed illustration of the need of knowledge in first aid in the case of snake bites and cholera. The Rev. H. J. Molony (C.M.S., India) said he felt the importance of the training to be acquired at Livingstone College to be so great that he was spending his furlough there. Mr. F. W. Savage (Assam) said that natives expected that white men should have some knowledge of medicine; whilst Mr. J. H. Lorrain (Assam) declared that there were two classes of missionaries who would be benefited by a course of instruction at Livingstone College—the first, who thought they were "glorifying God by taking no medicine; the second, who religiously swallowed a dose twice a day and wondered how it was that the climate did not agree with them!" Both became chronic invalids and a burden to their society, and some paid for their ignorance with their lives.

